# The invertebrate paleontology collection at Rio de Janeiro's Museu Nacional: museological practices and stages in its trajectory

**DOI:** 10.1590/S0104-59702024000100047en

**Published:** 2024-10-11

**Authors:** Joana D. Lima

**Affiliations:** i Researcher, Centro de Humanidades da Faculdade de Ciências Sociais e Humanas/Universidade Nova de Lisboa. Lisbon – Portugal joanalima@fcsh.unl.pt

**Keywords:** Cultural heritage in science and technology, History of collections, Museology, Paleontology collections, Museu Nacional (Rio de Janeiro

## Abstract

This article examines the general characteristics of how the invertebrate paleontology collection at the National Museum [Museu Nacional] in Rio de Janeiro took shape and the stages in its trajectory, considering the institution’s own journey from the perspective of museology and heritage studies. It addresses the reach of the collection within the context of the department and the procedures and practices involved, namely research, education, and exhibitions. The collection’s trajectory reflects the contexts that formed the backdrop for the museum, domestically and globally, between the mid-1900s and the early twenty-first century.

Understanding the creation and trajectory of the invertebrate paleontology collection within the Department of Geology and Paleontology [Departamento de Geologia e Paleontologia, DGP] at the National Museum (Museu Nacional), was the main objective of a broader investigation that developed a method to analyze this collection (Lima, 2019), organizing it into 71 subcollections and three other groups.^1^ The organization and grouping of data for each of the subcollections and groups made it possible to trace the trajectory of the collection as it intersected with that of the National Museum, marked by changes in areas that included authority, direction, space and organizational structure. This article describes this process and connects it to the institutional surroundings. To what degree are the transformations that occurred in the museum reflected in the trajectory of the collection? And conversely, what was the role of the collection in the institutionalization of paleontology at the museum?

Throughout nearly all of the nineteenth century, the National Museum was one of the few Brazilian institutions entirely dedicated to natural history; it was a pioneer in Brazilian contributions to the development of paleontology in the country. In this sense, it is important to understand the importance that invertebrate paleontology took on at the museum. When did the collection gain relevance in the department? How was it presented to the public? What concerns are inherent to exhibiting the specimens? What other activities was it involved in?

Various studies have addressed the National Museum, from historical documents produced by its directors (Lacerda, 1905; Netto, 1870; Duarte, 2019) and its institutional history (Duarte, 2019) to research on the preservation of memory and trajectory of its leadership (Benchimol et al., 2003; Lopes, 2008; Dau, 2008). There are also several disciplinary studies that help us understand the museum’s role in the institutionalization of science in Brazil, through integrated approaches in comparative studies (Lopes, 1997; Figueirôa, 1998; Considera, 2015) as well as more specific investigations, most notably in areas such as anthropology (Santos, 1998; Souza et al., 2009; Aranha Filho, 2011; Keuller, 2012), biology (Duarte, 2010), paleontology (Fernandes et al., 2007, 2017), or even on the role of certain researchers in the area of geology (Fernandes et al., 2010, 2011; Kunzler et al., 2011; Silva et al., 2013). Other studies focus on the importance of the museum’s publications in the promotion of science (Silva, 2012), the relevance of public courses during the nineteenth century (Sá et al., 1996), or the creation of the collections (Nascimento, 2009; Sá et al., 2008). In terms of museology, there are studies that allow us to understand certain aspects of the National Museum’s trajectory (Bessa, 2017) or address specific objects (Silva, 2010; Moreno Rocha, 2018), but none are directly related to paleontology.

This study is intended to fill this gap by analyzing the invertebrate paleontology collection from a museological perspective, with the National Museum demonstrating a limited expression of a broader phenomenon of the museum. Four areas of activity are most notable in the institution’s trajectory: (1) acquisition and handling of the collections; (2) teaching; (3) research; and (4) communications. Additionally, studying a museum collection implies exploring the stages of musealization which were involved: preservation, research, and communication (with a focus on exhibitions). This article explores how these functions dialog with the collection’s process of musealization.

Analysis of the discourse (via its regulations and statutes) and practices (via reports)^2^ carried out throughout the trajectory of the National Museum allow us to comprehend how its mission solidified. It provided the resources to identify the specific details of the collection, in line with the unique characteristics of the museum (typical of natural history museums) associated with a nation-building project (Schwarcz, 2012) and later incorporated into the Federal University of Rio de Janeiro [Universidade Federal do Rio de Janeiro, UFRJ].

The geographic reach and recognized scientific and historical value of this collection make this study an important contribution to research on museology, heritage, and the history of science. Furthermore, the value it adds is even more relevant after the fire that damaged the National Museum in September 2018.

## A collection under construction: the National Museum’s first (invertebrate) fossils

Looking back to the nineteenth century, the first fossils recorded as entering the collection date back to 1836 and correspond to a subcollection of specimens from the Piedmont region of Italy, sent to the museum by the paleontologist Giovanni Michelotti (1814-1898) (Michelotti, 24 jul. 1836; Relação..., 10 jan. 1837). There is another subcollection of fossils from the Paris basin, presented to King Pedro (Catalogue..., 30 jan. 1872) and subsequently donated to the museum in 1891 (Fernandes et al., 2008).

There is also a group of foreign fossils (corresponding to 24% of the collection and a total of 1,753 records) from Europe, North and South America, and Australia; how these specimens were incorporated into the collection is not clear. They most likely came from the Museum of Comparative Zoology at Harvard University, like another subcollection identified with this provenance that arrived at the National Museum via Charles Frederick Hartt (1840-1878), who completed his studies at that institution in Massachusetts and maintained relationships with Brazil by participating in expeditions.

The first subcollection of Brazilian fossils known to enter the collection was that of the Imperial Geological Commission (1875-1878), one of the most relevant not only for its size (1,332 record numbers) but also its historic and scientific values, as it represents one of the first efforts to identify Brazil’s invertebrate paleofauna.

The difficulty in finding information that can shed light on the formation and trajectory of the collection contrasts with the importance that the invertebrate fossils took on as early as the 1850s, with the first call for attention to foraminifera fossils:

We should not lose sight of demand for fossils of any nature, particularly foraminifera and infusoria, which often represent a very important role in the petrography of a country, and also perfectly characterize the formations in which they are found (Relatório..., 1857, p.4).^3^

In fact, there is a disconnect between the position paleontology occupied in the museum^4^ in the nineteenth century and the existence of records in the collection from that era. This resulted not only from the loss of documentation and specimens, but very likely also the evolution of invertebrate paleontology itself within the department compared to other more privileged areas such as minerology. The quantity of specimens for which no documentation was found and (inversely) the lack of correspondence between the data gathered and the collection itself suggest that samples collected up to the mid-1920s existed and would now be part of the collection but for various reasons did not survive to the present day.

Because of the interest they represented to science, explorations conducted by scientific commissions were part of the government’s priorities during the 1850s. They were one way for the museum to receive duplicates of collections and undertake studies it was unable to finance on its own (Derby, 13 maio 1886). Aligned with this interest was the appeal made by the director at that time, the engineer Frederico Leopoldo Burlamaque (1803-1866), to the various technical commissions that traversed the country to collect and send rocks, minerals, and fossils to the museum in line with the “Instructions on the preparation and shipping of the collections which were sent to them” (Instruções..., 1890) drafted for this purpose (Relatório..., 1893-1894).

But since the museum did not depend only on the government to expand its collections (Relatório..., 12 jan. 1921, 30 jan. 1922), it needed to send out requests to individuals in the provinces and various locations pointing out the importance of sending in these objects (Relatório..., 1885, 1886, 1887, 1890). This control of explorations was intended to stop collections which had been formed from leaving the country, such as sending duplicates for example (Relatório..., 1893-1894, 1906-1907).

As with the fossil subcollection of the Imperial Geological Commission (1875-1878), there may have been others with similar provenance; if not subcollections, at least pertinent data that guided the collection and entry of invertebrate fossils into the National Museum. The Imperial Scientific Commission (1859-1861) is such an example. Although paleontology was not its main focus, the commission produced positive scientific work in the collection of fossils (including shells) on the border with Piauí (Lopes, 2009). But a considerable part of the material collected by the Commission’s Geological Division was ultimately lost on the return to Rio de Janeiro when the boat carrying the collection, the *Palpite*, sank (Figueirôa, 2009).

Similarly, the invertebrate paleontology collection may have received fossils from the Imperial General Survey Committee (1862-1878), led by Charles Frederic Hartt, considering that paleontology and paleobotany (Vergara et al., 2011) were part of the Imperial geological research plan. The collections were known to have been transferred to the National Museum, and were inventoried by the geologist Orville Adalbert Derby (1851-1915) (Aviso..., 28 ago. 1878; Derby, 10 fev. 1879).

In 1879 Orville A. Derby was already heading the department and accompanied the Hydrographic Commission to conduct studies on the São Francisco River (Ofício..., 28 jul. 1879). The geological descriptions of the banks of this river contain references to the fossils that would have comprised a subcollection (Derby, 12 set. 1897), but none of the three record numbers associated with the geologist have this provenance. Years later, in 1886, Orville A. Derby agreed to lead another commission, this time to conduct a geographic and geological survey of the province of São Paulo (Aviso..., 5 maio 1886).

Expanding the petrology and paleontology collections was part of the department’s agenda, and is reflected in its interest in enriching them with objects collected by the Brazilian Coal Mine Study Commission (1904-1906), for example, which was led by the American geologist Israel Charles White (1848-1927) and also included museum staff (Relatório..., 28 jan. 1905). The commission’s report mentions an “interesting probe” conducted between 1856 and 1862 by Angelo Cassapis. Although no fossils were preserved (Relatório..., 9 dez. 1949), the collection does hold a record with this provenance (MN5065-I) which is associated with that collector.

The following should be mentioned:

It was in paleontology that the Museum was enriched the most during the year that ended, not only due to the quantity and quality of the samples (since unfortunately the regions accessible to our investigations have thus far proven to be notably poor in fossils), but also for the scientific importance of the few fossils received. Among these, a small collection brought from Mato Grosso by the American naturalist Herbert Smith deserves special mention (Parecer, 5 fev. 1885).

In fact, the only fossils in the collection, which were collected by Herbert Huntingdon Smith (1851-1919), come from Mato Grosso.

In line with this motivation was the need to hire a traveling naturalist to conduct geological and mineral exploration in the field (along with chemical analyses of rocks and minerals), in contrast with the “unfairness” of how staff were distributed across the different divisions of the museum, with the department suffering from the lack of a staff member to carry out these tasks (Parecer, 5 fev. 1885). This same need was mentioned again in subsequent years (Relatório..., 3 fev. 1902; Ofício..., 3 fev. 1947), and even though excursions were necessary not only to obtain specimens for trade as well as for the natural order of research, it was only overcome in the 1950s, and was most likely associated with the National Museum’s incorporation into the University of Brazil [Universidade do Brasil].

The period spanning 1888 to 1916 may be the time during which the National Museum underwent the most transformations since its creation. The adjustments during this phase, which correspond to not only the most decreed reforms but also reports, are reflected in the maturation of the institution resulting from a century of experience in an attempt to formalize what was best suited to its needs. One moment that was important to the history of the collection is when the facilities moved to Quinta da Boa Vista during June and July of 1892 (Relatório..., 31 dez. 1892). Descriptions of the state of the collections in the old building (which required conservation) as well as their transport suggest that the move did not have immediate benefits for the collection. The need for construction efforts to transform some halls of the palace (which took place in the following years) reflects a scenario in which invertebrate fossils were destroyed and lost.

The urgent need to reorganize and classify the collections led the director at that time, João Batista de Lacerda (1846-1915), to call for an inventory of all the objects and specimens in the museum. As a result, the department tallied 2,338 acquisitions corresponding to 5,178 items in three areas: mineralogy, petrology, and paleontology (Inventário..., 30 jul. 1904). Because of how these numbers were presented, it was not possible to determine how many of these items corresponded to invertebrate fossils. Furthermore, the first reference to invertebrate fossils only appeared in 1925, in a ministry report.^5^

Following the Centennial of Brazil’s independence, which was the motive for excursions to collect information and collections for the museum, there was a considerable expansion in the invertebrate paleontology collection, making it the most complete of Brazil’s paleontology collections (Relatório..., 1925). Most likely, this substantial expansion drove a review of the material on exhibit (by Mathias de Oliveira Roxo of the Geological Service), and resulted in a catalog of the paleontology collections which ended in 1927, of which no record have been found.

Despite the difficulty finding documentation on how the invertebrate paleontology collection was created, relative to the nineteenth century, an environment clearly existed which encouraged the movement and incorporation of specimens. A few months before the fire in September 2018, the collection totaled over 11,000 records: most from Brazil, North America, and Europe, and mostly collected from the 1940s onward.

## Handling of the collection and activities involving it

From roughly the 1920s onwards, concerns with the handling and care of the collection become notably more evident. What is important now is understanding what aspects were considered by the department leadership.

Even before the museum was transferred to the Quinta da Boa Vista palace, when Orville A. Derby took over as director of the department (1879) the collections lacked space and specialist care as well as the scientific coordination essential for suitable public display. Compared with the mineral collections, of which only the Werner collection was exhibited, and even then “stored in old and unsuitable cabinets, … classified and labeled, but with nearly illegible labels not suitable for public display and not worthy of this collection” (Derby, 22 jan. 1881), the other collections were in even worse shape:

Thousands of still-valuable samples were piled up without any indications whatsoever of their nature or provenance, the labels having been lost or destroyed by the humidity or the rats and cockroaches. The small yet good collection of fossils that existed in the Museum had been classified without any indication in relation to its local origins or the geological site, and the labels were often switched. The collections of the former Geological Commission were stored in the drawers in which they had arrived from the headquarters of that commission, and even though they were only partially classified and in a temporary arrangement, they were in the best condition of the entire lot. ... Using some cabinets that were old and unsuited for this purpose, a paleontology collection was exhibited, arranged in the order of geological sites, in which the old fossil collections are conveniently mounted and labeled and a small portion of those of the Geological Commission [are] partly classified (Derby, 22 jan. 1881).

Mineralogy stood out in the department, not only for its exhibition area but also its commitment to installing a laboratory.

Faced with this scenario, the priorities of the department director centered around classifying new materials; this was a purely scientific effort that would bring credit to the museum, but additionally, delaying classification risked further damage (Derby, 22 jan. 1881). In the meantime, foreign naturalists and collectors could potentially publish their own descriptions of the collections from duplicates, diverting the credit due to the National Museum for assembling type specimens. From its early days it competed with other museums in Europe, not only in classifying its species by identification but also in verifying the nomenclature which was “often suspicious even in collections received from Europe” (Relatório..., 1837). There was an awareness of the speed of science and a desire to keep up that contributed to its development.

Together with this consciousness were also concerns about the security of the collections, which were visible not only in the ban on removing any object from the institution “except for scientific or industrial exhibitions” (Regulamento..., 25 abr. 1888, Art.18º), but also the need to control the entry and exit of objects through the record log (Regimento..., 4 out. 1890; Regulamento..., 26 dez. 1892). But given the scale of the collection, the specimen data were only entered into the logs in the 1940s (Fernandes et al., 2008), when they were first organized.

Over its lifetime, the collection was classified several times. The first known occasion was the classification of the fossils collected by the Imperial Geological Commission (1875-1878) by the Americans Edward Drinker Cope (1840-1897) and Charles Abiathar White (1826-1910). This work resulted in the description of over two hundred species which were new and mentioned in publications with images of the fossils^6^ (Derby, 21 jul. 1883). A few years after the museum moved to Quinta da Boa Vista in 1892, several of the department’s samples whose labels had been lost were reclassified, and damaged labels were replaced. At this time a large number of specimens donated by the emperor were also classified.

In the twentieth century, specimens were reclassified by geological era, separating the fossils from various locations according to each period (Relatório..., 20 jan. 1915), and the specimens that came from abroad were systematically reviewed, along with the identification and organization of various groups of fossils (Relatório..., 5 fev. 1934). The files in the three areas of the department were also revised (Boletim..., 5 maio 1939). This most likely was the only attempt at standardizing how the collections were handled in line with the other departments in the museum, considering that each department observed its own criteria prior to the fire. Finally, when the department was incorporated into the Graduate Program in the Department of Geosciences in 1970, the collection began to be the subject of ongoing reviews by the students, not only from that department but also the Graduate Program in Biology/Zoology at the National Museum.

Alongside reclassification (which is common in collections of this type), progress in the housing of the paleontology collections can also be seen slowly taking shape. Collection and handling were addressed jointly through instructions on how to select and package the rocks, minerals, and fossils designated for the National Museum, which were part of a wider-ranging document containing instructions for each of the museum’s divisions (Instruções..., 1890). Although they tended to focus on collecting rocks and minerals, among the three levels considered (selection, treatment, and transport of the samples) we should highlight some aspects related to selection, since they specifically mention fossils:

When fossils are found (stones representing the remains of animals or plants, impressions of shells, or the shells themselves), they should be removed with all due care from the rocks that contained them. In any event, the samples shall be numbered in the order in which they were deposited in the ground and shall be accompanied (whenever possible) by a description of the site or a sketch that permits comparison with corresponding samples in order to provide an idea of the site which they were part of (Instruções..., 1890, p.4-5).

With the transfer of the National Museum to Quinta da Boa Vista and subsequent efforts to adapt the building, housing the collection came to extend over several years. The department’s collections were shifted from the old central pavilion of the palace to the lateral halls, a move notable for reflecting the intention to carry out public instruction using the exhibitions (Derby, 14 jul. 1884). At this point, the cabinets dedicated to the paleontology collections were renovated and expanded in number; these collections were housed and ordered in accordance with the classification commonly used at that time (Relatório..., 24 jan. 1899, 12 fev. 1890). With regard to the arrangement of the hall:

At the right of the entrance was the Brazilian collection, greatly enriched by the specimens brought by the late professor Hartt, to whom the hall is dedicated, in addition to the magnificent logs and petrified wood fragments, amid which some rare items can be noted. ... At the left, in a hall of nearly the same dimensions, the foreign paleontology collection was installed, one of the richest that has appeared in our land. ... To better emphasize not only this but also the paleontology collection, countless tags and labels were acquired that call attention and provide knowledge to the public on the contents of the cabinets (Relatório..., 12 fev. 1890).

The exhibit halls worked as spaces to store and present the specimens; in other words, the exhibition itself served as a storage space, since most of the material (which was already numbered, classified, and labeled) was stored in drawers. The inverse of this practice can be seen in the modern storage areas which are open to visitation, with the exhibition revealing a visible portion of the technical reserve collections.

Years later, within the framework of “intuitive teaching” that the government planned to implement throughout the Republic (Relatório..., 12 jan. 1921), the National Museum sought to teach natural sciences and play an active role in overcoming the organizational failures in the natural history collections at teaching institutions by broadening its outreach program as well as creating collections to serve as models for school museums. At the department scale, this intention manifested in an inventory of all the permanent material prior to the drafting of a guide entitled “Evolution of the Earth’s structure and the geology of Brazil, seen through the collections of the National Museum” [“A evolução da estrutura da Terra e a geologia do Brasil, vistas através das coleções do Museu Nacional”] (Leme, 1924). The guide was intended to mediate for visitors as they directly observed rocks, minerals, and fossils (Relatório..., 30 jan. 1922), and recommended cabinets containing the invertebrate specimens representing various periods in geological history.

Educating the public through the exhibitions has been very much present throughout the museum’s trajectory, making it a pioneer in presenting collections, particularly in paleontology, as can be seen in the description of an exhibition created by the department:

This is an original collection of rock specimens, typical fossils, maps, and geological cross-sections and Brazilian bibliographic recommendations which have not yet been organized in any Brazilian scientific institution and which present visitors with the entirety of Brazilian geology, with its known divisions, typical fossils and rocks, and the main sites (Relatório..., 10 jan. 1936, p.2).

Indeed, there was a concern with calling visitors’ attention to the “most important facts and the uses or applications of the samples on exhibit” (Relatório..., s.d.).

The 1940s marks an important time in the collection’s trajectory. The paleontology collections were formally reorganized, records associated with the samples were created, and a catalog was organized. At this time, 1,152 samples were prepared, corresponding to the subcollection of foreign fossils. There was also a plan to create a standard collection of Brazilian paleontology, with material obtained through an exchange with the Geological Service (Relatório..., 6 jan. 1941). The inventory of the fossils continued in the following years, with identification and distribution of the specimens into a total of 314 drawers. Even so, the materials from Brazil still awaited identification (Relatório..., 3 fev. 1943).

The director of the department at that time, Emmanoel A. Martins, began to foresee that space would run out for the collections in the technical storage area. In his understanding,

the only thing that is permanent ... and always tends to increase, is the scientific materials that are and will always and continuously be collected in the field and brought into storage, where they will accumulate for some time until they are directed elsewhere. For this reason, a large space in this division should be set aside for the material in storage and certainly without negatively affecting the laboratories (Ofício..., 1 ago. 1945).

As a consequence of these concerns, a plan was made to reorganize the department’s collections which resulted in the creation of a logbook, housing the invertebrate collection according to numerical order, and cataloging the collection by three different record systems: numerical, systematic, and geographic chronology (Ofício..., 13 nov. 1951). This reorganization, in line with the museological view of collections preserved in museums, made it possible to find any item in the collection by the type of search utilized, and permitted optimal use of the available space. Another notable aspect is the elimination of material considered not to be of interest, principally due to the lack of provenance and, in turn, scientific value. This implies previous selection of what was to be incorporated into the collection (or not) that considered the scientific value, rarity, or even the beauty of the items (Ofício..., 24 jun. 1946).

Equally relevant in the collection’s trajectory is the “DGP/Collections” project begun in November 1988 to restructure and recover part of the National Museum’s collections (“Recovering 100 years of the history of paleontology” [“A recuperação de 100 anos de história da paleontologia”]) (Relatório..., 20 out. 1989). This involved qualitative and quantitative assessment, along with taxonomical review and update of the specimens. Additionally, the collection was housed and organized on new stands and in another space, separating the type specimens from the rest. This exhaustive survey made it possible to track the items, many of which had been lent out and never returned, leading to the reestablishment of contacts and even the recovery of some of these fossils. It also resulted in the updated publication of a catalog of illustrated type specimens and figurative fossils from the collection (Fernandes et al., 2001). Most notably, special attention was paid to the material from the Imperial Geological Commission, with most of its collections placed into the department’s paleontology, mineralogy, and petrology divisions.

A decade later in 1989, an arrangement was made with the Mineral Research and Resources Company [Companhia de Pesquisas e Recursos Minerais], which was charged with inserting all the information about the invertebrate paleontology collection into a digital data system, the Geological Data System’s PALE (for “Paleontological Analyses”). This was an innovative measure for that time, and the only Brazilian initiative integrating paleontological data between institutions. Other activities to digitize the collection included the creation of a standardized database using Microsoft Access in 2014 that included specific fields for each of the department’s collections (Santos et al., 2016). By June 2018, a total of 1,190 records had been created, roughly 10% of the collection, including part of the type specimens.

## Stages in the creation and trajectory of the invertebrate paleontology collection

Considering its establishment, the care and handling it received, and the activities in which it was involved, the invertebrate paleontology collection (as it was in the National Museum up to the time of the 2018 fire) essentially resulted from four different stages that defined its creation and trajectory within the institution.

### The early days of the collection (1818 until the mid-1920s)

Marked in the trajectory of the National Museum as a period of great expansion of its collections, the interval between its creation and the end of the nineteenth century did not have precisely these same repercussions for the invertebrate paleontology collection. In general, the department’s activities only began to gain speed when Orville A. Derby took over the directorship in 1879 and prioritized more scientific work associated with classification tasks. as well as the preservation and exhibition of the collections.

Mineralogy became a highlight of the department, likely associated with the industrial leap seen in Brazil between 1840 and 1870 focused on mining and metallurgy activities (Schwarcz, 2012), particularly in Rio de Janeiro, Minas Gerais, and São Paulo, activities in which the National Museum acted as a scientific consultant. In line with nearly all modern museums, like the Museum of Plants in Paris and the La Plata Museum in Argentina (Relatório..., 1910-1911), in the early 1920s the museum did not change the original aspects of its program but expanded the scope of its research and studies by creating four laboratories, including the mineral chemistry lab. The department conducted scientific and industrial research in mineralogy using spectrochemical laboratory analyses and petrography by examining the eruptive magma of the Serra do Mar mountain chain.

This period in the collection’s history is marked by the museum’s move to Quinta da Boa Vista. While this change was positive in that it expanded the space designated for exhibitions, allowing much of the collections to be seen by the public, it also led to the loss of invertebrate fossils and the information that accompanied them. This is the most probable explanation for the lack of data on the collection during this period.

Despite concerns with the acquisition and handling of the collections, along with the research and teaching activities done with their involvement, through exhibitions, it seems evident that the invertebrate fossils were not considered at the same priority level as mineralogy, petrology, or even vertebrate paleontology. This situation began to reverse only in the mid-1920s when the collection began to benefit from the focus on the handling of the paleontology collections in general.

### The collection takes off (mid-1920s to 1950)

The 1920s marked a new stage in the collection’s trajectory, as invertebrate paleontology blossomed in the department. This period is marked by a considerable expansion in the number of specimens. Invertebrate fossils also began to be differentiated in the official documentation.

Within the context of the museum’s scientific consulting, invertebrate paleontology began to stand out due to its association with the first explorations of fossil fuels, notably petroleum, which emerged during this period (Relatório..., 12 jan. 1921, 4 jan. 1923, 29 out. 1926). At the base of these discoveries is the importance of paleontology in stratigraphic identification of the geological sites, making the development of the invertebrate paleontology collection at least partially linked to economic and industrial interests.

At a time when the museum had recently (1931) passed into the guardianship of the Ministry of Education and Public Health, within the National Department of Teaching, it is not surprising that research and the formation of new collections played an important role. As a result, greater concern can be seen with the collections that were not exhibited and had accumulated to a significant degree. Within this context, storage and identification of the specimens was prioritized.

Probably associated with this change in oversight, the 1940s was a period of qualitative and quantitative advances for invertebrate paleontology at the museum. Formalizing the entry of fossils into the collection through the record book and storage in numerical order comprised the first major project to reorganize the collection, with repercussions extending up to the time of the fire. It involved aspects which had not yet been considered, and calls attention to relevant museological questions such as economy of space. With the tasks involved in organizing the collection complete, the collection of new items was planned in order to gather Brazilian materials in quantities sufficient to resume donations and exchanges.

The 1950s saw the fruits of the first significant change in the collection, with the development of other activities more associated with academic work that reverberated through the subsequent decades. In this sense, the trajectory of the collection can be considered to have taken shape in the early 1920s and continued to solidify up to the 1950s, when it was understood to have been relatively solidified in comparison with the other DGP collections.

### The first university phase of the collection (1951-1980)

During this phase, the National Museum was incorporated into the University of Brazil (1946), which was transformed into the Federal University of Rio de Janeiro in 1965.

While invertebrate paleontology flourished during the previous period, growth which translated into the reorganization of the collection, the 1950s and following decades marked the consolidation of the collection in the department, which was largely associated with the establishment of the Invertebrate Paleontology Laboratory (Relatório..., 1957). After this lab was created, it began to take over part of the department’s routine tasks of preparing slides and molds and restoring invertebrate fossils.

Within a context of stimulating research and outreach projects where the collections played a central role, the department became incorporated into the Graduate Program in the Department of Geosciences (1970). At this time, the career of naturalist began to transform into specialty posts in each of the sciences, such as geologists, for example. From here onward, the quantity of documents about the activities conducted by each researcher becomes very significant.

Greater detail and differentiation of the studies and research according to each department division can be seen; the reports follow the same standard and consecutively include handling of the collections, research, excursions, and publications. When observed in detail, the data obtained in the collection reflect the geographical and geological areas that were favored at that time.

This period is characterized by a substantial increase in collection, which prior to that time had been scarce due to lack of funds. Participation in conferences, congresses, and symposia intensified, also indicating growth in studies. Notable among the various activities were assistance to primary and secondary education, continuing contributions by lending educational collections and teacher training, assistance to post-secondary paleontology departments in university geological science programs, and organization traveling exhibitions as well as receiving visitors to the department (Relatório..., 31 dez. 1957). These aspects indicate that the following decades were a period of major activity in the area of invertebrate paleontology.

### The second university phase of the collection (1981-2018)

The second university phase of the collection took place in the 1980s, with a new generation of researchers leading curatorship. There was a significant change in how the collection was stored, which characterizes this stage as particularly focused on questions of preservation, control, and safety for the collection. All this took place alongside continuation of the research projects and fieldwork.

The 2000s brought a new reality for the collection: projects were undertaken to recover historical data in order to locate various items assumed to have gone missing. Positive outcomes from recent research as part of these efforts include the discovery of a donation of shells from the Paris basin, the identification (and in the case of the vertebrate fossils, recovery) of items sent by Giovanni Michelotti during the first half of the nineteenth century (Fernandes et al., 2010), and the identification of fossils collected as part of the Morgan expeditions (1970-1971). By the time of the final survey of the collection, a significant number of fossils had been lost, and some contacts had already been made to pursue recovery in some cases. Considering the fire that ravaged the National Museum in 2018, these missing items now may potentially return to the collection.

This period is also characterized by new ways of doing fieldwork, with taphonomy offering new alternatives for collection that favor different ways of gathering data and handling the material in the laboratory. Meanwhile, the Antarctic expeditions undertaken by department researchers enriched the collection with foreign fossils, which up to that time had only happened in isolated cases. From a museological perspective, this phase is especially relevant for the repatriation of the Kenneth Edward Caster (1908-1992) subcollection to Brazil, which permits more profound exploration of questions related to returning collections to their countries of origin. Also as part of museological practices, greater concern with the basic information attributed to the subcollections (collector, collection and entry dates, age, provenance, classification, and respective authorship) is visible.

## Final considerations

Information is scarce on invertebrate fossils until the mid-1920s. The lack of documentation as well as information in the record books and associated with the specimens themselves makes it difficult to fully understand the creation and trajectory of the collection up to that time. However, a dataset could be created that helps trace the development of paleontology at the National Museum and understand how the collection was formed as a result of decisions made, not only at the direction level but also by the department itself.

From its very beginning, the National Museum was seen to be in line with the practices pertaining to natural history museums of that time. While not always declared openly as invertebrate fossils, the documentation suggests an institutional dynamic in which the enrichment of the collections for scientific purposes prevailed. In this sense, the invertebrate paleontology collection (as it was prior to the fire) most likely contains only what survived, and for this reason does not offer a faithful portrait of how it was created or its trajectory.

Even the difficulties finding explicit references to invertebrate fossils (up to the mid-1920s) is partly and likely due to the fact that paleontology from its origins was located at the intersection between zoology and geology, although it was made subordinate to geology for historical reasons. In line with the richness that the twentieth century represents in the development of this science, the invertebrate paleontology collection began to assume more importance in the department as fossils gained more scientific, political, and economic importance in fuel exploration.

Invertebrate paleontology benefits from the importance given to the combined treatment of the department’s collections. Although the literature indicates that the collection was first organized in the 1940s, there is evidence prior to this time indicating concerns with the classification, conservation, and exhibition of the collections, directly associated with research and teaching activities. These aspects combine to embody the museum’s overall lines of activity on the one hand, while they also are each suited to the associated functions of preservation, research, and communication as a phenomenon/place/institution which apply to the sensory experience. All in all, the National Museum’s functions dialog with the process through which the collection was musealized.

Still, the shift from the scale of the museum to the context of the invertebrate paleontology collection shows a discrepancy is visible between intentions and practices, manifesting from early on in the individual functioning of each department without standardized handling of the collections. In this respect, it would be interesting to explore how the collection was impacted by the various disciplines associated with each museum director.

The overall institutional concerns with the collections were not always directly reflected in the collection, due not only to the lack of staff focusing on acquisition and handling of the specimens but also the lack of space to store them conveniently. Still, progress can be seen not only in fossil collection methods but also how they were stored in order to best take advantage of them for research and teaching.

The National Museum’s invertebrate paleontology collection, as of April 2017 (the date of the last consultation), was the result of various processes of formation, namely collections, donations, and exchanges which in broad terms took place from the 1940s and were the objects of various curators. The four stages that comprise its 181-year trajectory reflect changes every 30 years (except for the first, longer period), which generally means that there were modifications every two or three generations of curators/researchers that reflect different ways of seeing and dealing with the subcollections, thus leading to a non-homogeneous final result.

The institution’s trajectory involved changes in facilities, participation in exhibitions, as well as different processes in handling, reorganization, and storage, which together encompass management of available space, elimination of material no longer of interest, and implementing security measures. Together, these activities made it possible to investigate and understand the role of the museum in managing the collections.

By actively participating in scientific and museological activities, guided either by the transformations in the National Museum or by the circumstances inherent to the department, the invertebrate paleontology collection plays an important role in the institutionalization of paleontology in Brazil.

## NOTES

1 There were a total of 10,400 records as of April 2017 (when last consulted). This number corresponds to roughly sixty thousand items, since one sample attributed a single number (an aggregate of sedimentary rock, for example) could be the source of multiple fossils.

2 Besides the collection, documents were also consulted: (1) in the Memory and Archives Division of the National Museum (SEMEAR); (2) in the DGP Invertebrate Paleontology Division; and (3) in the Central Library of the National Museum. This work with primary sources also included ministry reports (a total of 77) which are available online via the Center for Research Libraries.

3 In this and other citations of texts from Portuguese, a free translation has been provided.

4 Paleontology is included in the organizational structure of the museum, in the 1876 statutes, and is divided into three areas – animal, vegetable, and general – each belonging to a distinct section (Regulamento..., 9 fev. 1876).

5 One of the difficulties of identifying invertebrate fossils in the documentation lies in the generic mention of shells or shell collections, which could be included in malacology (within zoology) or also considered fossils. In this regard, at the time when Emmanoel Azevedo Martins, a naturalist in the department, also occupied the post of interim head of the museum’s malacology division, he transferred to this division three current collections of shells and mollusks (Naturalista..., 11 ago. 1942) which had entered as part of an exchange with the Baron Onoratto de Fiore di Cropani, a professor at the University of São Paulo (Fernandes et al., 2006).

6 John Mason Clarke (1857-1925) also described over fifty species left in his care by Orville A. Derby (Derby, 9 maio 1891).

## References

[B1] ARANHA Jaime Moraes (2011). Guia da impermanência das exposições: uma investigação sobre transformações do Museu Nacional do Rio de Janeiro nos anos 1940.

[B2] AVISO n.66 (1886). Diretoria, pasta 25, doc.66.

[B3] AVISO n.27 (1878). Diretoria, pasta 17, doc.79.

[B4] BENCHIMOL Jaime Larry (2003). Bertha Lutz e a construção da memória de Adolpho Lutz. História, Ciências, Saúde - Manguinhos.

[B5] BESSA Simone (2017). Musealização e ordenamento jurídico do museu no Brasil: missão e função (conceito e prática) no Museu Nacional/UFRJ (séculos XIX-XX)..

[B6] (1939). BOLETIM mensal de abril, 1939, Relatórios MN, Classe 146.1, Geologia e Mineralogia, caixa 1.

[B7] (1872). CATALOGUE des fossiles du bassin de Paris. DGP, caixa 1, dossiê 3.

[B8] CONSIDERA Andréa Fernandes (2015). Uma história dos fazeres museais no Brasil entre a segunda metade do século XIX e as primeiras décadas do século XX: Museu Nacional, Museu Paraense Emílio Goeldi, Museu Paranaense e Museu Paulista.

[B9] DAU Leda, Azevedo Nara, Cortes Bianca Antunes, Sá Magali Romero, Cortes Bianca Antunes (2008). Um caminho para a ciência: a trajetória da botânica Leda Dau. História, Ciências, Saúde - Manguinhos.

[B10] DERBY Orville A (1897). Carta ao diretor do Museu Nacional, Ladislau de Souza Melo Neto. Diretoria, pasta 18, doc.113.

[B11] DERBY Orville A (1891). Carta ao diretor do Museu Nacional, Ladislau de Souza Melo Neto. Diretoria, pasta 30, doc.60.

[B12] DERBY Orville A (1886). Carta ao diretor do Museu Nacional, Ladislau de Souza Melo Neto. Diretoria, pasta 25, doc.61.

[B13] DERBY Orville A (1884). Carta ao diretor do Museu Nacional, Ladislau de Souza Melo Neto. Diretoria, pasta 23, doc.121.

[B14] DERBY Orville A (1883). Carta ao diretor do Museu Nacional. Diretoria, pasta 22, doc.105.

[B15] DERBY Orville A (1881). Carta ao diretor do Museu Nacional, Ladislau de Souza Melo Neto. Diretoria, pasta 20, doc.13.

[B16] DERBY Orville A (1879). Carta ao diretor do Museu Nacional. Diretoria, pasta 18, doc.19.

[B17] DUARTE Luiz Fernando Dias (2019). O Museu Nacional: ciência e educação numa história institucional brasileira. Horizontes Antropológicos.

[B18] DUARTE Regina Horta (2010). A biologia militante: o Museu Nacional, especialização científica, divulgação do conhecimento e práticas políticas no Brasil, 1926-1945.

[B19] FERNANDES Antônio Carlos Sequeira (2017). Dalla nostra terra: as contribuições "geognósticas" italianas ao Museu Nacional.

[B20] FERNANDES Antônio Carlos Sequeira, Fiolhais Carlos (2011). José da Costa Azevedo e Custódio Alves Serrão: da formação na Universidade de Coimbra à importante atuação na estruturação do Museu Nacional no Brasil.

[B21] FERNANDES Antônio Carlos Sequeira, Brandão José Manuel (2010). Coleções e museus de geologia: missão e gestão.

[B22] FERNANDES Antônio Carlos Sequeira (2008). D. Pedro II, os fósseis da bacia de Paris e o Museu Nacional. Filosofia e História da Biologia.

[B23] FERNANDES Antônio Carlos Sequeira (2007). Histórico da paleontologia no Museu Nacional. Anuário do Instituto de Geociências.

[B24] FERNANDES Antônio Carlos Sequeira (2006). Os fósseis estrangeiros da coleção de paleoinvertebrados do Museu Nacional.

[B25] FERNANDES Antônio Carlos Sequeira (2001). Catálogo de fósseis-tipo e figurados da coleção de paleoinvertebrados do Museu Nacional Publicações Avulsas do Museu Nacional.

[B26] FIGUEIRÔA Silvia Fernanda de Mendonça, Kury Lorelai (2009). Comissão Científica do Império: 1859-1861.

[B27] FIGUEIRÔA Silvia Fernanda de Mendonça (1998). Mundialização da ciência e respostas locais: sobre a institucionalizaçã o das ciências naturais no Brasil (de fins do século XVIII à transiçã o ao século XX).. Asclepio.

[B28] (1890). INSTRUÇÕES sobre a preparação e remessa das coleções que lhe forem destinadas.

[B29] (1904). INVENTÁRIO dos objetos e espécimes existentes na 3ª Seção do Museu no dia 30 de julho de 1904 mandado fazer pelo diretor, o cidadão dr. João Batista Lacerda, para servir de base ao catálogo do museu, segundo a circular do mesmo cidadão de 25 de fevereiro de 1904.

[B30] KEULLER Adriana Tavares do Amaral Martoms (2012). Os estudos físicos de antropologia no Museu Nacional do Rio de Janeiro: cientistas, objetos, ideias e instrumentos (1876-1939).

[B31] KUNZLER Josiane (2011). Herbert Huntington Smith: um naturalista injustiçado?. Filosofia e História da Biologia.

[B32] LACERDA João Batista de (1905). Fastos do Museu Nacional do Rio de Janeiro.

[B33] LEME Alberto Betim Paes (1924). A evolução da estrutura da Terra e a geologia do Brasil, vistas através das coleções do Museu Nacional.

[B34] LIMA Joana David Caprário de (2019). A coleção de paleoinvertebrados do Museu Nacional do Rio de Janeiro (UFRJ): formação, trajetória e utilização em contexto museológico.

[B35] LOPES Maria Margaret, Kury Lorelai (2009). Comissão Científica do Império: 1859-1861.

[B36] LOPES Maria Margaret (2008). Proeminência na mídia, reputação em ciências: a construção de uma feminista paradigmática e cientista normal no Museu Nacional do Rio de Janeiro. História, Ciências, Saúde – Manguinhos.

[B37] LOPES Maria Margaret (1997). O Brasil descobre a pesquisa científica: os museus e as ciências naturais no século XIX..

[B38] MICHELOTTI Giovani (1836). Carta ao diretor do Museu Nacional.

[B39] MORENO ROCHA Saulo (2018). Esboços de uma biografia de musealização: o caso da Jangada Libertadora.

[B40] NASCIMENTO Fátima Regina (2009). A formação da Coleção de Indústria Humana no Museu Nacional, século XIX.

[B41] (1942). NATURALISTA interino. Divisão de Geologia e Mineralogia. Carta ao diretor do Museu Nacional.

[B42] NETTO Ladislau (1870). Investigações históricas e científicas sobre o Museu Imperial e Nacional do Rio de Janeiro: acompanhadas de uma breve notícia de suas coleções e publicadas por ordem do Ministério da Agricultura.

[B43] OFÍCIO n.697 (1951). Diretoria, Livros D143-144.

[B44] OFÍCIO n.6 (1947). Diretoria, DGP, caixa 3.

[B45] OFÍCIO s.n (1946). Diretoria, DGP, caixa 3.

[B46] OFÍCIO n.53 (1945). Diretoria, DGP, caixa 4.

[B47] OFÍCIO n.97 (1879). Diretoria, pasta 18, doc.97.

[B48] PARECER (1885). Diretoria, pasta 24, doc. 15A.

[B49] (1890). REGIMENTO de 1890, Decreto n.810.

[B50] (1892). REGULAMENTO de 1892. Decreto n.1.179.

[B51] (1888). REGULAMENTO de 1888. Decreto n. 9.942.

[B52] (1876). REGULAMENTO de 1876. Decreto n.6.116.

[B53] (1837). RELAÇÃO de conchas vivas, fluviais e terrestres e fósseis dos arredores de Roma remetidas ao gabinete de História Natural do Rio de Janeiro.

[B54] (1989). RELATÓRIO n.7 do Projeto Coleções/DGP, DGP.

[B55] (1957). RELATÓRIO do 2º semestre de 1957.

[B56] (1957). RELATÓRIO anual. Universidade do Brasil, Museu Nacional.

[B57] (1949). RELATÓRIO anual de 1949. Relatórios Museu Nacional, Classe 146.1, Geologia e Mineralogia, caixa 2.

[B58] (1943). RELATÓRIO da DGM de 1942. Relatórios MN, Classe 146.1, Geologia e Mineralogia, caixa 1.

[B59] (1941). RELATÓRIO da 1ª Seção de 1940. Relatórios MN, Classe 146.1, Geologia e Mineralogia, caixa 1.

[B60] (1936). RELATÓRIO da 1ª Seção de 1935. DGP, caixa 8.

[B61] (1934). RELATÓRIO anual de 1933. Relatórios MN, Classe 146.1, caixa 1.

[B62] (1926). RELATÓRIO da 1ª Seção de 1923 a 1926. DGP, caixa 5.

[B63] (1925). RELATÓRIO da Secretaria de Estado da Agricultura, Indústria e Comercio – Seaic.

[B64] (1923). RELATÓRIO da 1ª Seção de 1922. DGP, caixa 4.

[B65] (1922). RELATÓRIO da 1ª Seção de 1921. DGP, caixa 4.

[B66] (1921). RELATÓRIO da 1ª Seção de 1920. DGP, caixa 4.

[B67] (1915). RELATÓRIO da 3ª Seção de 1914. Diretoria, pasta 72, doc.21.

[B68] RELATÓRIO da Secretaria de Estado da Agricultura, Indústria e Comércio – Seaic, 1910-1911.

[B69] RELATÓRIO do Ministério da Justiça e dos Negócios Interiores, 1906-1907.

[B70] (1905). RELATÓRIO da 3ª Seção de 1904. Diretoria, pasta 46, doc.5.

[B71] (1902). RELATÓRIO da 3ª Seção de 1901. Diretoria, pasta 42, doc.1ª.

[B72] (1899). RELATÓRIO da 3ª Seção de 1898. Diretoria, pasta 37, doc.249.

[B73] do Ministério da Justiça e dos Negócios Interiores.

[B74] (1892). RELATÓRIO da 3ª Seção de 1892. Diretoria, pasta 31, doc.142.

[B75] (1890). RELATÓRIO da 3ª Seção de 1899. Diretoria, pasta 38, doc.222.

[B76] (1890). RELATÓRIO da Secretaria de Estado da Agricultura, Indústria e Comercio – Seaic.

[B77] (1887). RELATÓRIO da Secretaria de Estado da Agricultura, Indústria e Comercio – Seaic.

[B78] (1886). RELATÓRIO da Secretaria de Estado da Agricultura, Indústria e Comercio – Seaic.

[B79] (1885). RELATÓRIO da Secretaria de Estado da Agricultura, Indústria e Comercio – Seaic.

[B80] (1857). RELATÓRIO da Secretaria de Estado dos Negócios do Imperio – Seni. Instruções para a comissão científica encarregada de explorar o interior de algumas províncias do Brasil.

[B81] (1837). RELATÓRIO da Secretaria de Estado dos Negócios do Imperio – Seni.

[B82] RELATÓRIO da 1ª Seção de 1934. Relatórios MN, Classe 146.1, Geologia e Mineralogia, caixa 1.

[B83] SÁ Guilherme José da Silva e (2008). Crânios, corpos e medidas: a constituição do acervo de instrumentos antropométricos do Museu Nacional na passagem do século XIX para o XX. História, Ciências, Saúde – Manguinhos.

[B84] SÁ Magali Romero (1996). O Museu Nacional e o ensino de ciências naturais no Brasil no século XIX. Revista da Sociedade Brasileira de História da Ciência.

[B85] SANTOS Márcia F.A (2016). Uso do MS Access como aplicativo para base de dados no gerenciamento de coleções: estudo de caso em museus de paleontologia. Gaea: Journal of Geoscience.

[B86] SANTOS Ricardo Ventura (1998). A obra de Euclides da Cunha e os debates sobre mestiçagem no Brasil no início do século XX: “Os sertões” e a medicina-antropologia do Museu Nacional. História, Ciências, Saúde – Manguinhos.

[B87] SCHWARCZ Lilia Moritz (2012). A construção nacional: 1830-1889.

[B88] SILVA Marina Jardim (2013). Silva Coutinho: uma trajetória profissional e sua contribuição às coleções geológicas do Museu Nacional. História, Ciências, Saúde – Manguinhos.

[B89] SILVA Paulo Vinícios Aprígio da (2012). Nas páginas o que está escrito?: Os “Archivos do Museu Nacional” e a promoção das ciências no oitocentos.

[B90] SILVA Sabrina Damasceno (2010). “O pedaço de outro mundo que caiu na Terra”: as formações discursivas acerca do meteorito de Bendegó.

[B91] SOUZA Vanderlei Sebastião de (2009). Arquivo de Antropologia Física do Museu Nacional: fontes para a história da eugenia no Brasil. História, Ciências, Saúde – Manguinhos.

[B92] VERGARA Moema de Rezende (2011). A Comissão da Carta Geral do Império (1862-1878) e sua participação no contexto da cartografia brasileira no Império.

