# Supplementary material for: The invertebrate paleontology collection at Rio de Janeiro's Museu Nacional: museological practices and stages in its trajectory
Source: Hist Cienc Saude Manguinhos. 2024 Oct 11;31:e2024047. doi: 10.1590/S0104-59702024000100047en (PMC11472779; doi:10.1590/S0104-59702024000100047en)
Supplement: Translation

# Translation: A coleção de paleoinvertebrados do Museu Nacional, Rio de Janeiro: práticas museológicas e etapas da sua trajetória

**DOI:** 10.1590/S0104-59702024000100047

**Authors:** Joana D. Lima

**Affiliations:** i Investigadora, Centro de Humanidades da Faculdade de Ciências Sociais e Humanas/Universidade Nova de Lisboa. Lisboa – Portugal joanalima@fcsh.unl.pt

**Keywords:** Patrimônio cultural de ciência e tecnologia, História das coleções, Museologia, Coleções de paleontologia, Museu Nacional (Rio de Janeiro

## Abstract

O artigo analisa os aspetos gerais da formação da coleção de paleoinvertebrados do Museu Nacional, no Rio de Janeiro, e as etapas da sua trajetória, tendo em consideração o próprio percurso da instituição, sob a perspetiva da museologia e dos estudos sobre o património. São abordados o alcance da coleção no contexto do departamento, os procedimentos por que passou e as práticas em que esteve envolvida, nomeadamente a pesquisa, o ensino e as exposições. A trajetória da coleção reflete os contextos em que o museu se moveu no país e no mundo, desde meados do século XIX até o início do século XXI.

Compreender o processo de formação e a trajetória da coleção de paleoinvertebrados, inserida no Departamento de Geologia e Paleontologia (DGP) do Museu Nacional, vinculado à Universidade Federal do Rio de Janeiro (UFRJ), foi o objetivo central de uma investigação mais ampla que teve por base a criação de um método de análise desta coleção (Lima, 2019), o qual permitiu organizá-la em 71 subcoleções e três outros conjuntos.^1^ Com as informações organizadas e distribuídas por cada uma das subcoleções e conjuntos, foi possível traçar a trajetória da coleção na sua interseção com a trajetória do Museu Nacional, marcada por mudanças de tutela, direção, espaço, estrutura organizacional, entre outras. O objetivo deste artigo é relatar esse processo e relacioná-lo com o seu entorno institucional. Em que medida as transformações ocorridas no museu se refletem na trajetória da coleção? Inversamente, qual o papel da coleção na institucionalização da paleontologia no museu?

Por quase todo o século XIX, o Museu Nacional foi uma das poucas instituições brasileiras dedicadas primordialmente à história natural, tendo sido pioneiro na contribuição brasileira para o desenvolvimento da paleontologia no país. Nesse sentido, importa compreender a importância que a paleontologia de invertebrados foi assumindo no museu. Quando é que a coleção ganha relevância no departamento? Como foi sendo apresentada ao público? Quais as preocupações inerentes à exposição dos espécimes? Em que outras atividades participou?

São diversos os estudos sobre o Museu Nacional, desde documentos históricos produzidos pelos diretores (Lacerda, 1905; Netto, 1870; Duarte, 2019) e sobre a história institucional (Duarte, 2019), passando por estudos sobre a preservação da memória e a trajetória de quem o dirigiu (Benchimol et al., 2003; Lopes, 2008; Dau, 2008). Existem também vários estudos disciplinares que ajudam a compreender o papel que o Museu Nacional desempenhou na institucionalização das ciências no país, seja por meio de abordagens integradas em estudos comparativos (Lopes, 1997; Figueirôa, 1998; Considera, 2015), ou mais específicas, nomeadamente em áreas como a antropologia (Santos, 1998; Souza et al., 2009; Aranha Filho, 2011; Keuller, 2012); a biologia (Duarte, 2010); a paleontologia (Fernandes et al., 2007, 2017), ou até mesmo sobre o papel de determinados pesquisadores da área da geologia (Fernandes et al., 2010, 2011; Kunzler et al., 2011; Silva et al., 2013). Existem ainda estudos focados na importância das publicações do museu na promoção das ciências (Silva, 2012), na relevância que os cursos públicos tiveram no século XIX (Sá et al., 1996), ou mais focados na constituição de acervos (Nascimento, 2009; Sá et al., 2008). Sob o viés da museologia existem estudos que permitem compreender determinados aspetos da trajetória do Museu Nacional (Bessa, 2017) ou focados em objetos específicos (Silva, 2010; Moreno Rocha, 2018), mas nenhum diretamente relacionado com a paleontologia.

O presente estudo procura suprir essa ausência ao analisar a coleção de paleoinvertebrados sob a perspetiva da museologia, que encontra no Museu Nacional a expressão limitada do museu enquanto fenómeno mais amplo. Na trajetória da instituição sobressaem essencialmente quatro linhas de atuação: (1) aquisição e tratamento das coleções; (2) ensino; (3) pesquisa e (4) comunicação. Além disso, o estudo de uma coleção museológica implica conhecer as etapas da musealização por que passou – preservação, pesquisa e comunicação (com enfoque nas exposições). Este artigo procura compreender como é que aquelas funções dialogam com o processo de musealização da coleção.

A análise dos discursos (feita por meio dos regulamentos e regimentos) e das práticas (por meio dos relatórios)^2^ desenvolvidos na trajetória do Museu Nacional permitiu compreender como sua missão se foi consolidando. Formou o quadro necessário para identificar as especificidades da coleção, atendendo às características particulares do museu, tipicamente de história natural, associado a um projeto de construção da nação (Schwarcz, 2012) e, mais tarde, incorporado à UFRJ.

A abrangência geográfica e o reconhecido valor científico e histórico dessa coleção fazem desse estudo um importante contributo para as pesquisas em museologia, património e história das ciências. Além disso, o valor agregado é ainda mais relevante, depois do incêndio ocorrido em setembro de 2018.

## Uma coleção em formação: os primeiros fósseis (invertebrados) no Museu Nacional

Recuando ao século XIX, os primeiros fósseis de que há registo de entrada na coleção remontam a 1836 e correspondem a uma subcoleção de espécimes provenientes de Itália (Piemonte), enviada ao museu pelo paleontólogo Giovanni Michelotti (1814-1898) (Michelotti, 24 jul. 1836; Relação..., 10 jan. 1837). Há uma outra subcoleção de fósseis da bacia de Paris, oferecidos a dom Pedro (Catalogue..., 30 jan. 1872) e doados pelo imperador ao museu, em 1891 (Fernandes et al., 2008).

Existe ainda um conjunto de fósseis estrangeiros (corresponde a 24% da coleção, perfazendo um total de 1.753 registros) provenientes da Europa, das Américas do Norte e do Sul e da Austrália do qual se desconhece a forma como foram incorporados à coleção. Muito provavelmente vieram do Museu de Zoologia Comparada da Universidade de Harvard (Massachussetts), à semelhança do que aconteceu com a subcoleção identificada com essa proveniência, que terá chegado ao museu por intermédio de Charles Frederick Hartt (1840-1878), o qual completou os estudos naquela instituição e manteve relações com o Brasil ao participar em expedições.

Sabe-se ainda que a primeira subcoleção de fósseis brasileiros a dar entrada na coleção foi a da Comissão Geológica do Império (1875-1878), uma das mais relevantes não só pela dimensão (1.332 números de registo) como pelo seu valor histórico e científico, na medida em que representa os primeiros trabalhos de identificação de paleofaunas de invertebrados do Brasil.

A dificuldade em encontrar informações que permitam trazer à luz a formação e a trajetória da coleção contrasta com a importância que os fósseis invertebrados já assumiam na década de 1850, à qual remonta a primeira chamada de atenção para os fósseis foraminíferos:

Não se deve perder de vista a procura de fósseis de qualquer natureza que sejam, principalmente foraminíferos e infusórios, que muitas vezes representam um papel tão importante na petrografia de um país, além de caracterizarem perfeitamente as formações em que são encontrados (Relatório..., 1857, p.4).

Há, de fato, um desfasamento entre o lugar que a paleontologia ia ocupando no museu,^3^ no século XIX, e a existência de registos na coleção que remontem àquela época. Isso deve-se não somente a perdas de documentação e de espécimes, mas, muito provavelmente, à própria evolução da paleontologia de invertebrados no departamento, quando comparada às outras áreas mais privilegiadas de que é exemplo a mineralogia. A quantidade de espécimes sobre os quais não foi encontrada qualquer documentação e, inversamente, a falta de correspondência entre as informações reunidas e a coleção propriamente dita alargam a possibilidade de terem existido exemplares coletados até meados da década de 1920, que hoje fariam parte da coleção, mas, por diversos motivos, não chegaram à atualidade.

Pelo interesse que representavam para a ciência, as explorações desenvolvidas pelas comissões científicas tomaram parte das prioridades do governo na década de 1850. Constituíam uma forma de o museu receber duplicatas das coleções e empreender estudos para os quais não possuía verba (Derby, 13 maio 1886). Alinhado com esse interesse estava o apelo feito pelo então diretor, o engenheiro Frederico Leopoldo Burlamaque (1803-1866), às várias comissões técnicas que percorriam o território para coligirem e enviarem ao museu amostras de rochas, minerais e fósseis, tendo como guia as “Instruções sobre a preparação e remessa das coleções que lhe forem destinadas” (Instruções..., 1890) organizadas para o efeito (Relatório..., 1893-1894).

Porém, como o museu não dependia somente do governo para aumentar as suas coleções (Relatório..., 12 jan. 1921, 30 jan. 1922), era necessário enviar circulares apelando aos particulares das províncias e de diversas localidades para a importância do envio de objetos (Relatório..., 1885, 1886, 1887, 1890). Esse controlo das explorações tinha como objetivo impedir a saída do país das coleções formadas, por exemplo, com o envio de duplicatas (Relatório..., 1893-1894, 1906-1907).

Nesse sentido, tal como a subcoleção de fósseis da Comissão Geológica do Império (1875-1878), poderão ter existido outras com proveniências equivalentes. Senão subcoleções, pelo menos dados pertinentes que terão conduzido a coletas e à entrada de fósseis invertebrados no Museu Nacional. A Comissão Científica do Império (1859-1861) é exemplo disso. Embora a paleontologia não fosse o foco principal, poderiam surgir bons trabalhos científicos da coleta de fósseis (incluindo conchas) na fronteira com o Piauí (Lopes, 2009). Porém, grande parte do material coletado pela Seção Geológica da Comissão acabou por perder-se no regresso ao Rio de Janeiro, no naufrágio do navio Palpite (Figueirôa, 2009).

Do mesmo modo, é possível que a coleção de paleoinvertebrados tenha recebido fósseis da Comissão da Carta Geral do Império (1862-1878), dirigida por Charles Frederic Hartt, considerando que a paleontologia e a paleobotânica (Vergara et al., 2011) faziam parte do plano de estudo geológico do Império. Sabe-se ainda que as coleções foram transferidas para o Museu Nacional, ficando a inventariação ao cuidado do geólogo Orville Adalbert Derby (1851-1915) (Aviso..., 28 ago. 1878; Derby, 10 fev. 1879).

Em 1879, já à frente da direção do departamento, Orville A. Derby acompanhou a Comissão Hidrográfica, com o objetivo de realizar estudos no rio São Francisco (Ofício..., 28 jul. 1879). Nas descrições geológicas das margens desse rio encontra-se a referência a fósseis que terão constituído uma subcoleção (Derby, 12 set. 1897). Porém, nenhum dos três números de registo associados ao geólogo tem aquela proveniência. Anos mais tarde, em 1886, Orville A. Derby aceitou chefiar outra comissão, desta vez com o intuito de fazer o levantamento da carta geográfica e geológica da província de São Paulo (Aviso..., 5 maio 1886).

O aumento das coleções petrográficas e paleontológicas fazia parte da agenda do departamento e reflete-se, por exemplo, no interesse em enriquecê-las com as coletas feitas pela Comissão de Estudos das Minas de Carvão de Pedra do Brasil (1904-1906), liderada pelo geólogo norte-americano Israel Charles White (1848-1927) e na qual participaram funcionários do museu (Relatório..., 28 jan. 1905). No relatório daquela comissão, é mencionada uma “interessante sondagem” realizada entre 1856 e 1862 por Angelo Cassapis. Apesar de não ter sido conservado nenhum fóssil (Relatório..., 9 dez. 1949), existe na coleção um registo (MN5065-I) com esta proveniência, associado àquele coletor.

Vale ainda mencionar o seguinte:

Foi na paleontologia que o Museu mais se enriqueceu durante o ano findo, não tanto pelo número e perfeição das amostras (porque infelizmente as regiões acessíveis às nossas investigações têm provado até agora notável pobreza de fósseis), mas pela importância científica dos poucos fósseis recebidos. Entre estes, merece especial menção uma pequena coleção trazida de Mato Grosso pelo naturalista norte-americano Herbert Smith (Parecer, 5 fev. 1885).

De fato, os únicos fósseis existentes na coleção, coletados por Herbert Huntingdon Smith (1851-1919), são provenientes de Mato Grosso.

Em linha com essa motivação está a necessidade de contratar um naturalista viajante que pudesse fazer explorações geológicas e mineralógicas no campo (além de análises químicas em rochas e minerais), contrariada pela “injustiça” na distribuição do pessoal pelas diferentes seções do museu, na qual o departamento saía prejudicado pela falta de um empregado que se ocupasse daquelas tarefas (Parecer, 5 fev. 1885). Essa necessidade continuou a ser mencionada em anos seguintes (Relatório..., 3 fev. 1902; Ofício..., 3 fev. 1947) e, apesar das excursões serem essenciais não só para obter espécimes para troca como também para o curso natural das pesquisas, só veio a ser superada na década de 1950, muito provavelmente associada à incorporação do Museu Nacional à Universidade do Brasil.

O período de 1888 a 1916 é talvez aquele em que o Museu Nacional passou por mais transformações desde a sua criação. Os reajustes feitos nessa fase, à qual corresponde o maior número de reformas decretadas, mas também de relatórios, refletem um amadurecimento da instituição em virtude de um século de experiência, procurando formalizar o que melhor se adequava às suas necessidades. Um momento relevante para a história da coleção é a mudança das instalações para a Quinta da Boa Vista, entre junho e julho de 1892 (Relatório..., 31 dez. 1892). As descrições do estado em que se encontravam as coleções no antigo edifício – necessitadas de cuidados de conservação – e do transporte a que foram sujeitas, sugerem que aquela transferência não teve benefícios imediatos para a coleção. A necessidade de obras de transformação de alguns salões do palácio, que foram sendo realizadas nos anos seguintes, reflete um cenário pautado pela destruição e perda de fósseis invertebrados.

A urgência em reorganizar e classificar as coleções levou o então diretor, João Batista de Lacerda (1846-1915), a apelar à realização de um inventário de todos os objetos e espécimes existentes no museu. Como resultado, o departamento contabilizou 2.338 entradas correspondentes a 5.178 exemplares das três áreas: mineralogia, petrografia e paleontologia (Inventário..., 30 jul. 1904). Em função da forma como são apresentados esses valores, não é possível apurar quantos desses exemplares correspondiam a fósseis invertebrados. Além disso, só em 1925 é registada a primeira referência a invertebrados fósseis num relatório ministerial.^4^

Na sequência do Centenário da Independência, que motivou a realização de excursões para coletar informações e coleções para o museu, houve um aumento considerável da coleção de paleoinvertebrados, tornando-a, dentro das coleções de paleontologia, a mais completa do Brasil (Relatório..., 1925). Provavelmente, essa ampliação substancial impulsionou a revisão do material exposto (elaborada por Mathias de Oliveira Roxo, do Serviço Geológico), tendo como resultado um catálogo das coleções de paleontologia terminado em 1927, do qual não foi encontrado nenhum registo.

Apesar da dificuldade em encontrar documentação sobre a formação da coleção de paleoinvertebrados, relativa ao século XIX, é possível apontar para a existência de um ambiente propício à movimentação e à incorporação de espécimes. Poucos meses antes do incêndio ocorrido em setembro de 2018, a coleção somava mais de 11 mil números de registo com procedência sobretudo do Brasil, da América do Norte e da Europa, a maioria dos quais coletados a partir da década de 1940.

## Tratamento da coleção e atividades em que esteve envolvida

A partir da década de 1920, começa a ser mais evidente a preocupação com o tratamento da coleção. Importa agora compreender que aspetos foram considerados por parte de quem dirigiu o departamento.

Ainda antes da transferência do museu para o Palácio da Quinta da Boa Vista, quando Orville A. Derby assume a direção do departamento (1879), depara-se com um cenário onde prevaleciam a falta de espaço para as coleções, bem como a ausência de tratamento por um especialista e de coordenação científica indispensáveis para expô-las devidamente ao público. Comparadas com as coleções mineralógicas, da qual a coleção Werner era a única exposta – e, ainda assim, “guardada em armários velhos e não adequados, … classificada e rotulada, porém, com os rótulos quase ilegíveis, impróprios à exposição pública e indignos dessa coleção” (Derby, 22 jan. 1881) –, as demais coleções encontravam-se ainda em piores circunstâncias, descritas da seguinte forma:

Milhares de amostras, aliás valiosas, estavam amontoadas sem indicação alguma quanto à sua natureza ou procedência, os rótulos tendo sido perdidos ou destruídos pela humidade ou pelos ratos e baratas. A pequena, porém boa coleção de fósseis, que existia no Museu, estava classificada sem indicação alguma em relação à sua procedência local e ao terreno geológico e com os rótulos muitas vezes trocados. As coleções da extinta Comissão Geológica estavam guardadas nas gavetas em que tinham vindo da sede daquela comissão e, posto que somente em parte classificadas e n’um arranjo provisório, eram de todas as que se achavam em melhor estado. ... Utilizando alguns armários velhos e impróprios para o fim, tem-se exposto uma coleção paleontológica, arranjada na ordem dos terrenos geológicos, e na qual se acham as velhas coleções de fósseis convenientemente montadas e rotuladas e uma pequena porção das da Comissão Geológica, em parte classificada (Derby, 22 jan. 1881).

A mineralogia destacava-se no departamento, não só pela área de exposição que ocupava como pelo empenho na instalação de um laboratório.

Perante esse cenário, as prioridades do diretor do departamento centram-se na classificação do material novo, por se tratar de trabalho puramente científico que daria crédito ao museu, mas também por representar a tarefa com mais riscos de prejuízo pela demora (Derby, 22 jan. 1881). A possibilidade dos naturalistas e colecionadores estrangeiros publicarem as descrições das coleções feitas a partir de duplicatas, desviaria a instituição da vantagem de reunir fósseis-tipo. Desde cedo, o Museu Nacional competia com os demais museus europeus, não só na classificação das espécies por identificar, como na verificação da denominação “muitas vezes suspeita mesmo nas coleções recebidas da Europa” (Relatório..., 1837). Havia consciência da velocidade a que se produzia ciência e a vontade de acompanhá-la, contribuindo para o seu desenvolvimento.

Alinhada com essa tomada de consciência, estava a preocupação com a segurança das coleções, expressa não somente na proibição de retirar qualquer objeto da instituição, “salvo para exposições científicas ou industriais” (Regulamento..., 25 abr. 1888, Art.18º), mas também na necessidade de controlar a entrada e saída de objetos, por meio do livro de registros (Regimento..., 4 out. 1890; Regulamento..., 26 dez. 1892). No entanto, à escala da coleção, a inserção das informações sobre os espécimes nos livros de registo só acontece na década de 1940 (Fernandes et al., 2008), considerado o primeiro momento da sua organização.

Ao longo da sua trajetória, a coleção foi classificada diversas vezes. A primeira de que há conhecimento foi a classificação dos fósseis coletados pela Comissão Geológica do Império (1875-1878), realizada pelos norte-americanos Edward Drinker Cope (1840-1897) e Charles Abiathar White (1826-1910). Esse trabalho resultou na descrição de mais de duzentas espécies novas e mencionadas em publicações com imagens dos fósseis^5^ (Derby, 21 jul. 1883). Uns anos após a mudança das instalações do museu para a Quinta da Boa Vista, realizada em 1892, foi feita a reclassificação de diversas amostras do departamento, cujos rótulos se extraviaram, e a substituição daqueles que estavam danificados. Nessa época, foi também classificado um grande número de espécimes doados pelo imperador.

Já no século XX foi feita a classificação dos espécimes por idade geológica, separando para cada período os fósseis oriundos das diversas localidades (Relatório..., 20 jan. 1915); e a revisão sistemática dos espécimes com proveniência estrangeira, acompanhada da identificação e arrumação dos diversos grupos de fósseis (Relatório..., 5 fev. 1934). Houve ainda uma reforma nos fichários das três áreas do departamento (Boletim..., 5 maio 1939). Essa foi, muito provavelmente, a única tentativa de padronização do tratamento das coleções alinhada com os demais departamentos do museu, tendo em conta que cada departamento seguia, até à data do incêndio, os seus próprios critérios. Finalmente, quando o departamento passa a integrar o Programa de Pós-graduação do Instituto de Geociências, em 1970, a coleção começa a ser alvo permanente de revisões feitas pelos alunos, não só daquele como do Programa de Pós-graduação em Ciências Biológicas/Zoologia do Museu Nacional.

A par com a reclassificação, comum em coleções dessa natureza, lentamente começam a verificar-se progressos no acondicionamento das coleções de paleontologia. A coleta e o tratamento são tomados em conjunto por meio das instruções sobre o modo de escolher e acondicionar rochas, minerais e fósseis destinados ao Museu Nacional, inseridas num documento mais amplo que contempla instruções para cada uma das seções do museu (Instruções..., 1890). Apesar de estarem tendencialmente focadas na coleta de rochas e minerais, entre os três níveis considerados (seleção, tratamento e transporte das amostras) vale destacar alguns aspetos relacionados com a seleção, por mencionarem especificamente os fósseis:

Quando se encontrarem ... fósseis (pedras representando vestígios de animais ou de plantas; impressões de mariscos ou os próprios mariscos), devem ser retiradas com todo o cuidado, de par com as rochas que os contiverem. Em todo o caso as amostras serão numeradas na ordem em que estiverem colocadas no terreno e serão acompanhadas (sempre que possível for) de uma descrição do terreno ou de um esboço organizado de tal forma que da sua comparação com as amostras correspondentes, se possa ter ideia do terreno de que faziam parte (Instruções..., 1890, p.4-5).

Com a transferência do Museu Nacional para a Quinta da Boa Vista e as sucessivas obras de adaptação do edifício, o acondicionamento da coleção acabou por estender-se por vários anos. Vale assinalar a deslocação das coleções do departamento do antigo pavilhão central do palácio para as salas laterais, por refletir a intenção de desenvolver a instrução pública por via das exposições (Derby, 14 jul. 1884). Nessa altura, houve uma renovação e um aumento do número de armários destinados às coleções de paleontologia, ficando dispostas e ordenadas de acordo com a classificação vigente na época (Relatório..., 24 jan. 1899, 12 fev. 1890). A respeito da disposição da sala:

À direita da entrada ficou a coleção brasileira, bastante enriquecida pelos espécimes trazidos pelo finado professor Hartt, a quem foi dedicada a sala, acrescida pelos magníficos toros e fragmentos de madeiras fosseis petrificadas, entre as quais notam-se alguns exemplares raros. ... À esquerda, em sala quase das mesmas dimensões, foi instalada a coleção paleontológica estrangeira, que é das mais ricas que tem aparecido no nosso território. ... Para melhor salientar não só esta como a coleção paleontológica, foram adquiridos inúmeros rótulos e dísticos que chamassem a atenção e dessem conhecimento ao público do conteúdo dos armários (Relatório..., 12 fev. 1890).

As salas de exposição funcionavam como espaços de guarda e apresentação dos espécimes, ou seja, a própria exposição servia de depósito, na medida em que boa parte do material – numerado, classificado e etiquetado – estava guardado em gavetas. Essa prática encontra paralelo nas reservas visitáveis, características da atualidade, embora no sentido inverso, uma vez que a exposição revela práticas visíveis nas reservas técnicas.

Anos mais tarde, enquadrado nas bases do “ensinamento intuitivo” que o governo tencionava implantar em toda a República (Relatório..., 12 jan. 1921), o Museu Nacional procurou desenvolver o ensino das ciências naturais e ter um papel ativo na superação das falhas na organização de gabinetes de história natural nas instituições de ensino, quer por meio da ampliação do seu programa de divulgação, quer da formação de coleções que servissem de modelo aos museus escolares. À escala do departamento, essa intenção manifestou-se no inventário de todo o material permanente que antecedeu a elaboração de um guia sob o título: “A evolução da estrutura da Terra e a geologia do Brasil, vistas através das coleções do Museu Nacional” (Leme, 1924). O guia procurava mediar o visitante na observação direta de rochas, minerais e fósseis (Relatório..., 30 jan. 1922), com a indicação dos armários onde, entre outros, constavam os espécimes invertebrados representantes dos diversos períodos da história geológica.

O ensino do público por meio das exposições está bem presente na trajetória do museu, tornando-o pioneiro na apresentação de coleções, nomeadamente de paleontologia, como se pode constatar pela descrição de uma exposição elaborada pelo departamento:

Trata-se de uma coleção original de espécimes de rochas, fósseis típicos, mapas e cortes geológicos e indicações bibliográficas brasileiras, ainda não organizada em instituto científico algum do Brasil e que permitirá expor aos visitantes toda a geologia brasileira, com as suas divisões admitidas, com os fósseis e rochas típicos e com a extensão conhecida dos principais terrenos (Relatório..., 10 jan. 1936, p.2).

Havia, de fato, uma preocupação em chamar a atenção dos visitantes para “os fatos mais importantes e para as utilidades ou aplicações das amostras expostas” (Relatório..., s.d.).

A década de 1940 marca um momento relevante na trajetória da coleção. Foi feita uma reorganização formal das coleções de paleontologia, acompanhada da elaboração de fichas associadas aos exemplares e da organização de um catálogo. Nessa época, foram preparados 1.152 espécimes que correspondem à subcoleção de fósseis estrangeiros. Havia ainda a perspetiva de completar uma coleção-padrão de paleontologia do Brasil, com material obtido por permuta com o Serviço Geológico (Relatório..., 6 jan. 1941). O inventário dos fósseis continuou nos anos seguintes, com a identificação e distribuição dos espécimes por um total de 314 gavetas. Ainda assim, o material proveniente do Brasil ficou a aguardar identificação (Relatório..., 3 fev. 1943).

O então diretor do departamento, Emmanoel A. Martins, começa a prever a falta de espaço para as coleções na reserva técnica. No seu entender,

a única coisa que é permanente ... e tende sempre a aumentar, é o material científico que é e será, sempre e continuadamente, coletado no campo e trazido para depósito onde se acumulará por muito tempo até que lhe seja dado outro destino. Por isso, para o material de depósito, deveria ter sido destinado um grande espaço desta Divisão é certo que sem prejuízo dos laboratórios (Ofício..., 1 ago. 1945).

Na sequência dessas preocupações, foi elaborada uma proposta de reorganização das coleções do departamento, que resultou na criação de um livro de registos, no acondicionamento da coleção de paleoinvertebrados seguindo a ordem numérica, e na sua catalogação de acordo com três fichários distintos – numérico, sistemático e cronogeográfico (Ofício..., 13 nov. 1951). Essa reorganização, em linha com o olhar da museologia para as coleções preservadas em museus, viabilizou a localização de qualquer exemplar da coleção consoante o tipo de pesquisa e permitiu uma melhor gestão do espaço disponível. Outro aspeto que sobressai é a eliminação do material destituído de interesse, nomeadamente pela falta de proveniência e, consequentemente, de valor científico. Pressupõe uma seleção prévia do que deve ser incorporado, ou não, à coleção, tendo em consideração o valor científico, a raridade ou até mesmo a beleza dos exemplares (Ofício..., 24 jun. 1946).

Igualmente relevante na trajetória da coleção é o projeto “Coleções/DGP”, iniciado em novembro de 1988, com o objetivo de reestruturar e recuperar parte das coleções científicas do Museu Nacional (“A recuperação de 100 anos de história da paleontologia”) (Relatório..., 20 out. 1989). Sumariamente, foi feita uma avaliação qualitativa e quantitativa, acompanhada da revisão e atualização taxonómica dos espécimes. Além disso, a coleção foi acomodada e organizada em novos suportes e num outro espaço, com a separação dos fósseis-tipo dos restantes exemplares. Esse levantamento exaustivo permitiu rastrear exemplares, muitos deles emprestados e nunca devolvidos, tornando possível restabelecer contatos e inclusive recuperar alguns desses fósseis. Resultou ainda na publicação atualizada de um catálogo de fósseis-tipo e figurados da coleção (Fernandes et al., 2001). Em particular, foi dada especial atenção ao material proveniente da Comissão Geológica do Império, com grande parte das suas coleções depositadas nas seções de paleontologia, mineralogia e petrografia do departamento.

Uma década depois, em 1989, foi elaborado um convénio com a Companhia de Pesquisas e Recursos Minerais, que se encarregou de inserir todas as informações sobre a coleção de paleoinvertebrados num sistema digital de dados – o Pale (Análises Paleontológicas) do Sistema de Informações Geológicas. Trata-se de uma medida inovadora para a época, além de constituir a única iniciativa brasileira de integração de dados paleontológicos entre instituições. Ainda dentro das ações de informatização da coleção, em 2014 foi criado um banco de dados, utilizando o programa Microsoft Access, devidamente padronizado, com a inclusão de campos específicos para cada coleção do departamento (Santos et al., 2016). Até junho de 2018, tinham sido criados, no total, 1.190 registos, ou seja, cerca de 10% da coleção, incluindo uma parte dos fósseis-tipo.

## Etapas do processo de formação e trajetória da coleção de paleoinvertebrados

Considerando a sua formação, o tratamento por que passou e as atividades em que esteve envolvida, a coleção de paleoinvertebrados – tal como se encontrava no Museu Nacional até à data do incêndio, em 2018 – resulta essencialmente de quatro momentos que enquadram a sua formação e trajetória na instituição.

### Os primórdios da coleção (1818 até meados da década de 1920)

Marcado na trajetória do Museu Nacional como um período de grande ampliação das suas coleções, o intervalo que vai desde a sua criação até ao final do século XIX não teve exatamente as mesmas repercussões na coleção de paleoinvertebrados. Genericamente, as atividades do departamento só começam a ganhar ritmo quando Orville A. Derby assume a direção, em 1879, e prioriza o desenvolvimento do trabalho mais científico, associado às tarefas de classificação, além da conservação e exposição das coleções.

A mineralogia assumia lugar de destaque no departamento, muito provavelmente associado ao surto industrial que o Brasil experimentou entre 1840 e 1870, centrado nas atividades mineradoras e metalúrgicas (Schwarcz, 2012), em particular, no Rio de Janeiro, em Minas Gerais e São Paulo; atividades nas quais o Museu Nacional desempenhava um papel de consultoria científica. Seguindo a orientação de quase todos os museus modernos, como, por exemplo, o Museu das Plantas (Paris) e o Museu de La Plata (Argentina) (Relatório..., 1910-1911), na viragem para a década de 1920, sem alterar a feição original do seu programa, o museu ampliou a esfera de estudos e pesquisas com a criação de quatro laboratórios, incluindo o de química mineral. O departamento desenvolveu pesquisas de caráter científico e industrial nas áreas da mineralogia – por meio de análises laboratoriais espectroquimicas – e da petrografia, por intermédio do estudo dos magmas eruptivos da Serra do Mar.

Esse período da trajetória da coleção é marcado pela transferência do museu para a Quinta da Boa Vista. Se, por um lado, essa mudança foi benéfica, na medida em que aumentou o espaço destinado às exposições, permitindo que muitas das coleções passassem ao conhecimento público, por outro, terá conduzido à perda de fósseis invertebrados e das informações que os acompanhavam. Isso explica, muito provavelmente, a falta de dados sobre a coleção nesse período.

Apesar da preocupação com a aquisição e o tratamento das coleções, associados às atividades de pesquisa e ao ensino que se procurava desenvolver por meio das exposições, parece evidente que os fósseis invertebrados não estavam na mesma linha das prioridades dadas à mineralogia, à petrografia e até mesmo à paleontologia de vertebrados. Essa situação começa a reverter-se somente em meados da década de 1920, quando a coleção começa a beneficiar do foco dado ao tratamento das coleções de paleontologia em geral.

### O arranque da coleção (meados da década de 1920 até 1950)

A década de 1920 marca uma nova etapa na trajetória da coleção, caracterizada por um florescimento da paleontologia de invertebrados no departamento. Esse período é marcado por um aumento considerável do número de espécimes. Além disso, os fósseis invertebrados começaram a ser discriminados na documentação oficial.

No contexto da consultoria científica prestada pelo museu, a paleontologia de invertebrados começa a ganhar destaque pela sua associação às primeiras explorações de combustíveis fósseis, em particular de petróleo, que surgem nesse período (Relatório..., 12 jan. 1921, 4 jan. 1923, 29 out. 1926). Na base dessas descobertas está a importância da paleontologia para a identificação estratigráfica dos terrenos geológicos, pelo que, em parte, a formação da coleção de paleoinvertebrados está ligada a interesses económicos e industriais.

Numa altura em que a tutela do museu havia passado recentemente (1931) para o Ministério da Educação e Saúde Pública, inserido no Departamento Nacional de Ensino, não é de estranhar que a pesquisa e a formação de novas coleções ocupassem lugar de destaque. Consequentemente, observa-se uma maior preocupação com as coleções não expostas, que se iam acumulando significativamente. Nesse contexto, priorizam-se o acondicionamento e a identificação dos espécimes.

Provavelmente associada a essa mudança de tutela, a década de 1940 representa um salto qualitativo e quantitativo para a paleontologia de invertebrados no museu. A formalização da entrada dos fósseis na coleção, por meio do livro de registos, e o seu acondicionamento por ordem numérica constituem o primeiro grande projeto de reorganização da coleção, com repercussões até à data do incêndio. Abrange aspetos até então não considerados e chama a atenção para questões museológicas pertinentes, como a economia de espaço. Uma vez concluídas as tarefas de organização da coleção, previa-se a coleta de novos exemplares, procurando reunir material brasileiro em quantidade suficiente para poder retomar as doações e permutas.

A década de 1950 acaba por usufruir da primeira evolução significativa da coleção, com o desenvolvimento de outras atividades mais associadas ao trabalho académico, que acabam por respaldar-se nas décadas seguintes. Nesse sentido, pode-se dizer que a trajetória da coleção vem tomando forma no início da década de 1920 e vai-se firmando até à década de 1950, compreendida como um momento de consolidação relativamente às restantes coleções do DGP.

### A primeira fase universitária da coleção (de 1951 até 1980)

Esse período corresponde, genericamente, à primeira fase universitária da coleção, com a incorporação do Museu Nacional na Universidade do Brasil (1946), transformada em Universidade Federal do Rio de Janeiro, em 1965.

Se o período anterior é marcado pelo florescimento da paleontologia de invertebrados, traduzido na reorganização da coleção, a década de 1950 e as que se seguem, marcam a consolidação da coleção no departamento, em grande medida associada à montagem do Laboratório de Paleontologia de Invertebrados (Relatório..., 1957). Com a sua criação, passam a fazer parte dos trabalhos de rotina do departamento a preparação de lâminas, a moldagem e a restauração de fósseis invertebrados.

Num contexto de estímulo aos trabalhos de pesquisa e divulgação, onde as coleções assumem um papel central, o departamento passa a integrar o Programa de Pós-graduação do Instituto de Geociências (1970). Começa também a ser considerado o desdobramento da carreira de naturalista em cargos de especialidade para cada uma das ciências, onde surgem os geólogos, entre outros. A partir de então, é bastante significativa a quantidade de documentos sobre as atividades desenvolvidas por cada pesquisador.

Observa-se um maior detalhamento e a discriminação dos estudos e pesquisas para cada setor do departamento. Os relatórios seguem o mesmo padrão e contemplam consecutivamente o tratamento das coleções, as pesquisas, as excursões e publicações. Quando observados em detalhe, os dados recolhidos na coleção refletem as áreas geográficas e geológicas que foram sendo privilegiadas.

Esse período caracteriza-se por um aumento substancial das coletas, até então escassas por falta de verba. Intensifica-se a participação em conferências, congressos e simpósios, de resto, indicadores do aumento das pesquisas. De entre as diversas atividades realizadas, destacam-se: a assistência aos ensinos primário e secundário, dando continuidade ao envio de coleções didáticas e à formação de professores; a assistência ao ensino superior, por meio do auxílio às cadeiras de paleontologia das faculdades de ensino das ciências geológicas; a organização de exposições itinerantes, bem como o atendimento aos visitantes do departamento (Relatório..., 31 dez. 1957). Esses aspetos apontam para que as décadas seguintes representem um período de grande atividade na área da paleontologia de invertebrados.

### A segunda fase universitária da coleção (de 1981 até 2018)

A década de 1980 marca a segunda fase universitária da coleção, com outra geração de investigadores à frente da curadoria. Ocorre uma mudança considerável no seu acondicionamento, que caracteriza essa fase como particularmente focada nas questões de preservação, controle e segurança da coleção. Tudo isso em paralelo com a continuidade dos projetos de pesquisa e dos trabalhos de campo.

Os anos 2000 constituem uma nova realidade para a coleção. Começam a desenvolver-se projetos para recuperação de dados históricos, na tentativa de localizar diversos exemplares tidos como desaparecidos. São exemplos positivos desses desenvolvimentos os resultados obtidos com pesquisas recentes, nomeadamente: a descoberta da doação de conchas da bacia de Paris; a identificação (e no caso dos fósseis vertebrados, a recuperação) dos exemplares encaminhados por Giovanni Michelotti, na primeira metade do século XIX (Fernandes et al., 2010), e a identificação dos fósseis coletados no âmbito das Expedições Morgan (1970-1971). Até à data do último levantamento de dados na coleção, havia um número significativo de fósseis extraviados e alguns contatos já estabelecidos com vista à recuperação de parte deles. Tendo em conta a tragédia ocorrida em 2018 no Museu Nacional, esses exemplares desaparecidos têm agora a possibilidade de retornar à coleção.

Esse período também é caracterizado por novas formas de fazer campo, onde os estudos tafonómicos trazem outros procedimentos de coleta, que privilegiam diferentes metodologias de levantamento de dados e tratamento do material em laboratório. Por outro lado, as expedições à Antártica, com a participação de investigadores do departamento, enriqueceram a coleção com fósseis estrangeiros, situação que, até então, tinha ocorrido apenas pontualmente. Na perspetiva da museologia, essa fase é particularmente relevante pelo repatriamento da subcoleção Kenneth Edward Caster (1908-1992) para o Brasil, ao permitir aprofundar questões relacionadas com o retorno das coleções aos países de origem. Ainda dentro das práticas museológicas observa-se maior preocupação com as informações básicas atribuídas às subcoleções (coletor, datas de coleta e entrada, idade, proveniência, classificação e respectiva autoria).

## Considerações finais

São escassas as informações sobre fósseis invertebrados até meados da década de 1920. A falta de documentação, mas também de informação, nos livros de registo e associada aos próprios espécimes dificulta o conhecimento sobre a formação e a trajetória da coleção até àquele período. No entanto, foi possível reunir um conjunto de dados que ajudam a traçar o desenvolvimento da paleontologia no Museu Nacional e a compreender como a coleção se foi formando em função das decisões tomadas, tanto no nível da direção como também do próprio departamento.

Desde a sua origem, o Museu Nacional mostra-se alinhado com as práticas próprias dos museus de história natural da época. Embora nem sempre declaradamente mencionados como fósseis invertebrados, a documentação sugere uma dinâmica institucional na qual prevalecia o enriquecimento das coleções para fins científicos. Nesse sentido, a coleção de paleoinvertebrados, tal como se encontrava antes do incêndio, é, muito provavelmente, constituída apenas pelo que sobreviveu, e não o retrato fiel da sua formação e trajetória.

Mesmo a dificuldade em encontrar (até meado da década de 1920) referências explícitas a fósseis invertebrados deve-se muito provavelmente, e em parte, ao fato de a paleontologia se encontrar, desde cedo, na confluência ou dependência da zoologia e da geologia, embora subordinada à última por razões históricas. Alinhada com a riqueza que o século XX representa no desenvolvimento da paleontologia, a coleção de paleoinvertebrados começa a ganhar destaque no departamento, associada ao protagonismo científico, político e económico assumido pelos fósseis na exploração dos combustíveis.

A paleontologia de invertebrados beneficia da importância dada ao tratamento conjunto das coleções do departamento. Apesar de a bibliografia apontar a década de 1940 como o primeiro momento da organização da coleção, existem evidências anteriores a esse período que indicam uma preocupação com a classificação, conservação e exposição das coleções, diretamente associadas às atividades de pesquisa e ensino. No conjunto, esses aspetos englobam, por um lado, as linhas gerais de atuação do museu; por outro, enquadram-se nas funções de preservação, pesquisa e comunicação a ele associadas, enquanto fenómeno/lugar/instituição que pousa sobre a experiência sensível. Em suma, as funções do Museu Nacional dialogam com o processo de musealização da coleção.

Quando, no entanto, caminhamos da escala do museu para o contexto da coleção de paleoinvertebrados, há uma discrepância entre as intenções e as práticas, desde logo manifestada no funcionamento individual de cada departamento, sem uma padronização no tratamento das coleções. A esse respeito, seria interessante explorar o impacto na coleção das diversas áreas disciplinares que estiveram à frente do museu, na pessoa de cada diretor.

As preocupações gerais da instituição com as coleções nem sempre tiveram reflexo direto na coleção, não somente pela falta de funcionários focados na aquisição e tratamento dos espécimes, como também pela falta de espaço para acondicioná-los convenientemente. Não obstante, observam-se progressos não só nos métodos de coleta dos fósseis, como na forma de acondicioná-los com vista ao seu melhor aproveitamento para a pesquisa e o ensino.

A coleção de paleoinvertebrados do Museu Nacional, tal como se encontrava até abril de 2017 (data da última consulta), resulta de vários processos de formação, nomeadamente coletas, doações e permutas ocorridas, *grosso modo*, a partir da década de 1940, e foi alvo de várias curadorias. Nos seus 181 anos de formação e trajetória, as quatro etapas por que passou refletem mudanças sensivelmente a cada 30 anos (à exceção do primeiro período, mais longo), o que significa, *grosso modo*, que a cada duas ou três gerações de curadores/investigadores ocorrem modificações que refletem diferentes formas de olhar e tratar as subcoleções, pelo que o resultado final não é homogéneo.

A sua trajetória envolveu a mudança de instalações, a participação em exposições, além de diversos processos de tratamento, reorganização e acondicionamento que, no conjunto, abrangiam a gestão do espaço disponível, a eliminação do material destituído de interesse e a implementação de medidas de segurança. No conjunto, essas atividades permitiram compreender o papel do museu na gestão das coleções.

Ao participar ativamente em atividades científicas e museológicas, pautadas quer pelas transformações ocorridas no Museu Nacional quer pelas circunstâncias inerentes ao departamento, a coleção de paleoinvertebrados assume papel de destaque na institucionalização da paleontologia no Brasil.

## NOTAS

1 No total, corresponde a 10.400 números de registo, contados até abril de 2017 (data da última consulta). Esse número corresponde a cerca de sessenta mil exemplares porque, por vezes, de uma mesma amostra (aglomerado de rocha sedimentar) à qual é atribuída um número, podem ser extraídos um ou vários fósseis.

2 Além da coleção, foram consultados documentos: (1) na Seção de Meméoria e Arquivo do Museu Nacional (Semear); (2) na Seção de Paleoinvertebrados do DGP e (3) na Biblioteca Central do Museu Nacional. A consulta de fontes primárias incluiu também os relatórios ministeriais (77, no total), disponíveis *on-line* por meio do Center for Research Libraries.

3 A paleontologia é incluída na estrutura organizacional do museu no regulamento de 1876, sendo dividida em três áreas – animal, vegetal e geral –, cada qual pertencente a uma seção distinta (Regulamento..., 9 fev. 1876).

4 Uma das dificuldades em identificar os fósseis invertebrados na documentação está na menção genérica a conchas ou coleções conquiológicas, que tanto podem ser incluídas na malacologia (área da zoologia) como podem ser consideradas fósseis. A esse respeito, vale mencionar que, na época em que Emmanoel Azevedo Martins, naturalista do departamento, ocupava também o cargo de chefe interino do setor de malacologia do museu, transferiu para este setor três coleções de conchas e moluscos atuais (Naturalista..., 11 ago. 1942), que haviam entrado por permuta realizada com barão Onoratto de Fiore di Cropani, professor na Universidade de São Paulo (Fernandes et al., 2006).

5 Também John Mason Clarke (1857-1925) descreveu mais de cinquenta espécies deixadas a seu cuidado por Orville A. Derby (Derby, 9 maio 1891).
